# Visually driven chaining of elementary swim patterns into a goal-directed motor sequence: a virtual reality study of zebrafish prey capture

**DOI:** 10.3389/fncir.2013.00086

**Published:** 2013-05-10

**Authors:** Chintan A. Trivedi, Johann H. Bollmann

**Affiliations:** Neural Circuits and Behavior Group, Department of Biomedical Optics, Max Planck Institute for Medical ResearchHeidelberg, Germany

**Keywords:** zebrafish, prey capture, virtual reality, goal-directed behavior, intermittent locomotion, double-step saccade, motor sequence, saccadic suppression

## Abstract

Prey capture behavior critically depends on rapid processing of sensory input in order to track, approach, and catch the target. When using vision, the nervous system faces the problem of extracting relevant information from a continuous stream of input in order to detect and categorize visible objects as potential prey and to select appropriate motor patterns for approach. For prey capture, many vertebrates exhibit intermittent locomotion, in which discrete motor patterns are chained into a sequence, interrupted by short periods of rest. Here, using high-speed recordings of full-length prey capture sequences performed by freely swimming zebrafish larvae in the presence of a single paramecium, we provide a detailed kinematic analysis of first and subsequent swim bouts during prey capture. Using Fourier analysis, we show that individual swim bouts represent an elementary motor pattern. Changes in orientation are directed toward the target on a graded scale and are implemented by an asymmetric tail bend component superimposed on this basic motor pattern. To further investigate the role of visual feedback on the efficiency and speed of this complex behavior, we developed a closed-loop virtual reality setup in which minimally restrained larvae recapitulated interconnected swim patterns closely resembling those observed during prey capture in freely moving fish. Systematic variation of stimulus properties showed that prey capture is initiated within a narrow range of stimulus size and velocity. Furthermore, variations in the delay and location of swim triggered visual feedback showed that the reaction time of secondary and later swims is shorter for stimuli that appear within a narrow spatio-temporal window following a swim. This suggests that the larva may generate an expectation of stimulus position, which enables accelerated motor sequencing if the expectation is met by appropriate visual feedback.

## Introduction

Goal-directed behaviors consist of sequenced movements that bring the organism closer to a desired object, location or insight, typically associated with reward. The properties of the target, the sensory processing capabilities, and the architecture of the motor system determine whether the execution of movement steps is continuous or discrete in time. For instance reaching and smooth pursuit eye movements are classic examples when movement steps are combined fluently to generate a smooth trajectory (Wolpert and Ghahramani, 2000; Lisberger, 2010). On the other end of the spectrum, the class of chained, interrupted motor sequences is epitomized by saccadic eye movements (Land, 1999; Schall and Thompson, 1999), which steer our gaze during tasks such as visual search or reading a text.

A well-studied and paramount type of goal-directed motion is visually guided prey capture behavior, which involves the tracking and pursuit of a target typically moving in an unpredictable fashion. Thus, a substantial part of the visuomotor circuitry must be geared toward the efficient control of this behavior, which comprises the detection and classification of objects and the selection of appropriate motor patterns to approach and capture the prey (Ewert et al., 2001). In visually guided prey capture, the animal must solve the problem of reducing the angle between the target and its own heading direction while simultaneously approaching the target. Individual movement steps may be preprogrammed and executed ballistically; alternatively, continuous target tracking and pursuit movements may be adjusted in real time according to the changing trajectory of the prey. In both cases, visual feedback is essential for generating subsequent motor commands in order to correct for target displacement and motor errors.

A quantitative analysis of how the spatio-temporal properties of the stimulus impact such complex motor sequences can provide information about the underlying neural mechanisms, (e.g., Schlegel and Schuster, 2008). Approaches in which a restrained animal is presented with artificial stimuli in a closed-loop configuration have been developed in order to mimic the effect of the animal's own movement responses on sensory input [“virtual reality,” reviewed by Dombeck and Reiser (2012)]. These techniques enable the experimenter to sample the visuomotor system using precisely controlled stimulus sequences with expected or unexpected visual feedback, and also to measure underlying neural activity using opto- and electrophysiological techniques (Harvey et al., 2009; Dombeck et al., 2010; Seelig et al., 2010; Ahrens et al., 2012).

Larval zebrafish exhibit visually guided motor-behaviors beginning at 4 days post fertilization, including robust optomotor and optokinetic responses (reviewed in Neuhauss, 2003; Portugues and Engert, 2009; Fero et al., 2011). Notably, zebrafish also engage in prey capture behavior beginning around 5 days post fertilization. When hunting prey, the fish performs a number of approaching swimming maneuvers, interrupted by brief pauses (McElligott and O'Malley, 2005), characteristic of intermittent locomotion (Kramer and McLaughlin, 2001). When the prey is in striking distance, the fish performs a capture swim (Borla et al., 2002), which terminates the sequence. Fin-tail co-ordination during prey capture (McClenahan et al., 2012) as well as individual examples of these bout-like swim patterns have been described kinematically and subjected to a categorical description (Borla et al., 2002; McElligott and O'Malley, 2005; Bianco et al., 2011). Furthermore, prey capture behavior depends on vision, and ablation of the tectum and of tegmental projection neurons in the nucleus of the medial longitudinal fasciculus (nMLF) suggested that these anatomical structures are likely to be serial components in the visuomotor pathway mediating this behavior (Gahtan et al., 2005).

While individual swims during prey capture were observed to represent slow forward swims and unique low-angle turns exclusively performed during this behavior (“J-turns,” McElligott and O'Malley, 2005), less is known about how the entire motor sequence is assembled in time from individual swim patterns. For instance, it is unclear whether swim bouts occurring early and late during the prey capture sequence may represent a single class of elementary motor pattern that could be modulated on a continuous scale to cover a large range of turning angles. Furthermore, although single swims could be evoked in an open-loop assay using artificial stimuli (Bianco et al., 2011), it is unknown how visual feedback controls the timing of individual swim bouts within such a sequence, which requires closed-loop visual stimulation techniques not yet developed in this model system.

To address these questions, we used high-speed video to record prey capture sequences of freely moving larvae, which yielded a comprehensive overview of motor patterns used in this behavior. Importantly, we recorded full-length prey capture sequences in the presence of only one paramecium at a time, which allowed us to observe target-directed turning patterns in a large angular range in the absence of stimulus competition. A quantitative analysis of visual properties of the prey during this naturally occurring behavior was used to design a set of virtual prey stimuli that were able to trigger target-directed sequences in minimally restrained larvae in a closed-loop assay. Also, by introducing small perturbations of motor-induced visual feedback at high temporal and spatial resolution, we observed that the timing of motor output was dependent on the location and timing of visual feedback. Parts of this work have been reported in abstract form (Trivedi et al., 2011).

## Results

### Swim sequences during prey-capture behavior

**Figure 1:**
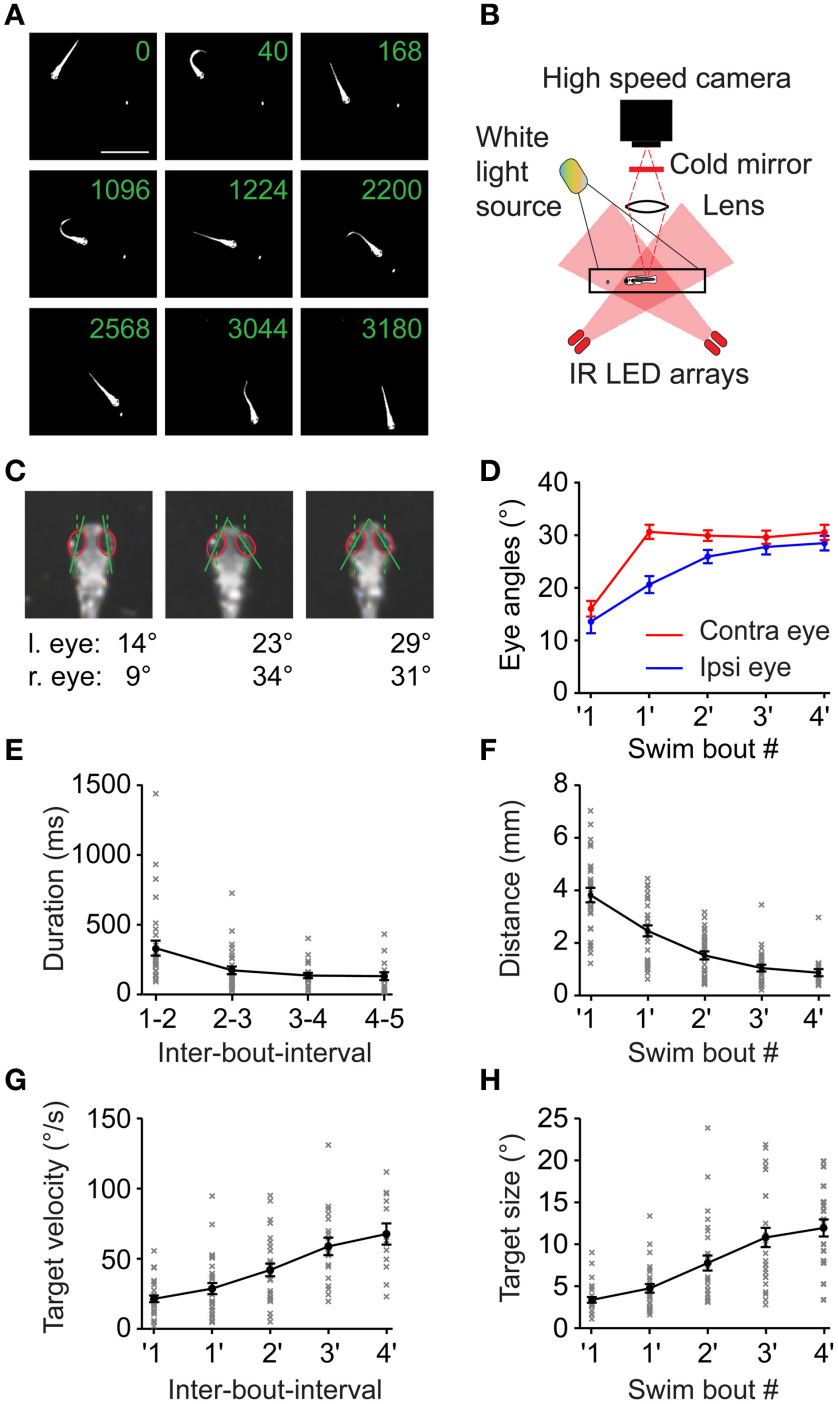
**Swim sequences during prey capture behavior. (A)** Selected frames of a 6 dpf larva performing a prey capture sequence recorded at 250 frames/s showing swim and rest episodes. Same field of view for all 9 frames (scale bar: 4 mm). Only one paramecium present (elongated white object, highlighted by local contrast enhancement). Numbers in each frame indicate time in milliseconds. Frames 2, 4, 6, and 8 show the 1st, 2nd, 4th and the capture swim in the sequence, respectively. **(B)** Experimental setup to record high speed movies of freely moving larva capturing prey. **(C)** Ipsilateral and contralateral eye angle measurements before 1st, after 1st and after 2nd swim. Magnified view of larval head, rotated to an upright position for clarity. Red ellipses: outline of the eyes, solid lines: major axis of ellipses; dashed lines: fish heading direction. **(D)** Ipsilateral and contralateral eye angles during the prey capture sequence (mean ± sem; *n* = 30 sequences). Note: eyes are specified as ipsilateral or contralateral based on the location of the prey target before the first swim of the sequence. This assignment was maintained for eye angle measurements made throughout the sequence irrespective of the location of the target in successive swims. **(E)** The interval between two successive swim bouts (IBI) decreased monotonically as the sequence progressed (*n* = 30 sequences). **(F)** Distance between the larva and the prey decreased monotonically with each swim from 3.8 ± 0.27 mm before first swim to 0.89 ± 0.13 mm after 4th swim. **(G)** Angular velocity of the prey measured between two swims increased monotonically from 21.1°/s ± 2.3°/s before first swim to 67°/s ± 7.5°/s after 4th swim. **(H)** Angular size of the prey increased from 3.2° ± 0.3° before first swim to 11.9° ± 1° after 4th swim. In **(D), (F), (G),** and **(H),** ′1 indicates measurements immediately before the first swim of a sequence, while 1′ indicates measurements immediately after the first swim and so on.

When swimming freely in a small arena to which a paramecium is added, larval zebrafish quickly engage in prey capture behavior. The larva performs several swim bouts within a few 100s of milliseconds, during which the larva successively minimizes the angle and distance between its body axis and the prey, stepwise approaching the prey until it is close enough to capture the object with high probability (Figure 1A) (McElligott and O'Malley, 2005).

Here, we use high-speed video recordings of zebrafish larvae performing full-length prey capture sequences in a small arena in the presence of single paramecia under ambient white light illumination (Figure 1B; Movies S1, S2). These movies were recorded using infrared dark-field illumination, which allowed us to record eye and tail movements and to measure the geometric relationships between hunter and prey in detail (Figures 1A,C). Prey capture sequences are interspersed with spontaneous swims at irregular intervals. We observed that the first prey-directed swims were accompanied by near maximal convergence of the eye contralateral to the prey, while the ipsilateral eye converged partially. Only the second swim brought both eyes into a maximally converged configuration (Figure 1D), which the fish maintained until after it had attempted a capture swim against the prey. We chose this characteristic two-step eye convergence pattern as a criterion to define the start of a prey capture sequence (Trivedi et al., 2011) (Figure 1C) in order to investigate the spatio-temporal dynamics of subsequent swims of this multistep motor behavior. Following the first swim in a sequence, the larva performed prey-directed swims in rapid succession. We analyzed 30 high-speed movies, in each of which an entire sequence from first swim to the final prey capture swim was recorded. These sequences had a mean duration of 1.23 ± 0.13 s (mean ± sem), and consisted of 4.4 ± 0.28 individual swim bouts (excluding the capture swim, *n* = 30 sequences). The inter-bout-interval (IBI) between swims decreased from 324 ± 54 ms after the first swim, to a minimum IBI of 124 ± 27 ms after the 4th swim (*n* = 30 sequences) (Figure 1E). The fish-object distance decreased monotonically (Figure 1F). Because only a single paramecium was present at all times, we could unambiguously determine the salient geometric features of the prey at the beginning and throughout the prey capture sequence. From measurements in single video frames immediately before and after a swim, we determined the angular size and angular velocity of the targeted paramecium relative to the midpoint between the eyes (Figures 1G,H). As expected from elementary geometry, average angular size, and velocity increased as the fish approached the prey.

### Single swims cover a large angular range, controlled by target position

**Figure 2:**
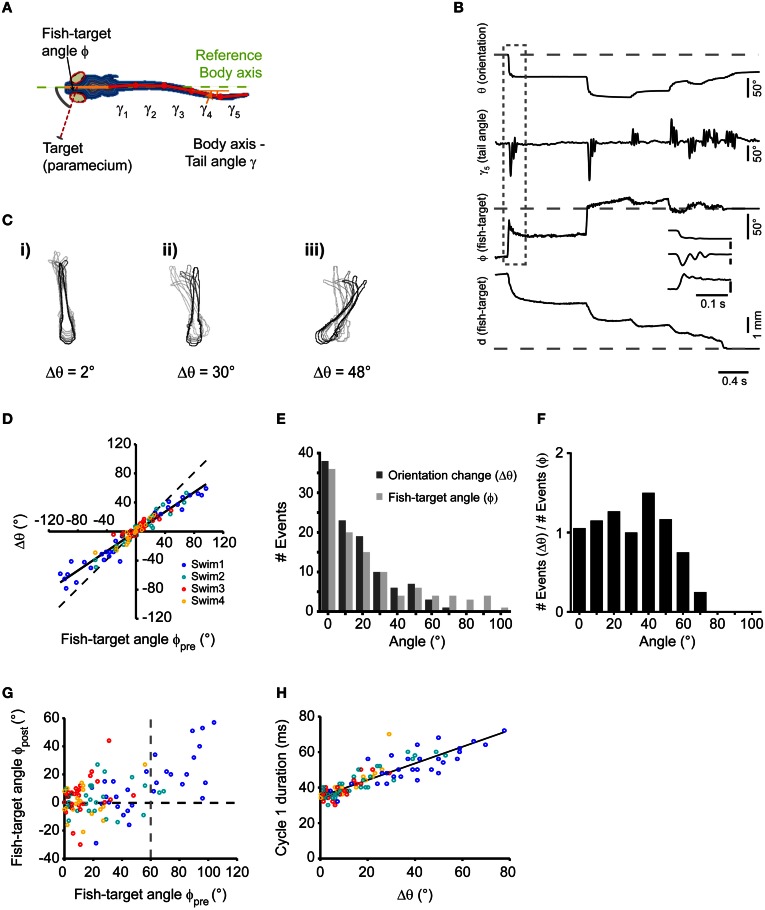
**Single swims cover a large angular range, controlled by target position. (A)** Illustration depicting automated image processing and parameter extraction from an individual frame. Colored contour lines represent distance map of the fish body. Six segments were used to fit the midline (Solid orange line: head segment; solid red lines: the tail split into five equidistant segments). Broken green line represents body axis with reference to which the deviations of the tail segments (γ_1_,…, γ_5_) were measured. **(B)** Time course of analyzed parameters for the sequence shown in Figure 1A on a frame-by-frame basis. Inset shows a blow up of the first swim bout of the sequence (dashed box). **(C)** Three examples (**i,ii,** and **iii**) of swims associated with change in orientation of 2, 30, and 48°, respectively. Every 10th frame (frame rate: 500/s) of the fish contour is overlaid. Light to dark contours indicate the progress of the swim bout from beginning to end. **(D)** Scatter plot of the change in body orientation (Δθ) generated by a swim bout vs. fish-target angle (ϕ_pre_) preceding the swim. Broken line: unity line; Solid line: straight line fit. **(E)** Histogram of fish-target angle (ϕ_pre_) and change in orientation (Δθ) from the data shown in **(D)**. Data were grouped into 10°-bins (1st bin contains angles from −5 to 5°, 2nd bin contains angles from −5 to −15° and from 5 to 15°, and so on). **(F)** Histogram of the orientation change (Δθ), normalized to the occurrence of fish-target angles (ϕ_pre_) for the data shown in **(D)**. Bin width as in **(E)**. **(G)** Scatter plot of fish-target angle after each swim (ϕ_post_) vs. fish-target angle before the swim (ϕ_pre_). Data points with negative fish-target angles (corresponding to prey on left side) were point-reflected about the origin. **(H)** Scatter plot of duration of first cycle in a swim bout vs. the resulting change in orientation. Data points with negative changes in orientation (corresponding to swims toward the left) were mirror-reflected about the y-axis. In **(D), (G),** and **(H)**, data points are color-coded to show the 1st, 2nd, 3rd, and 4th swims during a sequence.

Next, we analyzed the kinematics of individual swims during the prey capture sequence in order to relate swim output to visual input during individual steps of the motor sequence. Zebrafish larvae at this stage use tail beat frequencies between 20 and 80 Hz (Budick and O'Malley, 2000; McLean et al., 2008), which makes it difficult to measure tail kinematics and the relative timing of eye and tail movements with high precision when using frame rates ≤ 100 Hz (Bianco et al., 2011). Therefore, we used recordings at 250 or 500 Hz to measure tail and eye movements as a basis for kinematic analysis. Video records were analyzed automatically using a machine vision algorithm that determined the midline of the larva in each frame after binary operations and distance map conversion (Materials and Methods). The midline was then divided into six line segments consisting of the head segment, representing the body axis, and five tail segments (Figure 2A). The angular deviations γ_1_(*t*), …, γ_5_(*t*) of tail segments were automatically measured against the body axis. Furthermore, the angle ϕ(*t*) between the position of the prey with respect to the body axis and its distance *d*(*t*) with respect to the midpoint between the eyes were extracted automatically. Thus, the temporal evolution of the prey capture sequence was parameterized using a set of 8 observables (*d*, ϕ, θ, γ_1_, …, γ_5_) (Figures 2A,B).

Swim bouts during prey capture exhibited one-sided, asymmetric tail bending on a graded scale, which led to different degrees of turning, as shown in three examples in Figure 2C (i–iii). Among these swim bouts, we observed small angle turns that were not observed during spontaneous swimming, consistent with earlier results (McElligott and O'Malley, 2005). When comparing the change in orientation (Δθ) with the fish-target angle immediately before the swim (ϕ_pre_), a high correlation was observed (*r*_Pearson_ = 0.97, *p* < 10^−10^), which suggests that the fish can orient toward the prey on a fine graded scale (Figure 2D). The distribution of turning angles (Δθ) across all swims varied smoothly and corresponded well to the distribution of fish-target angles (ϕ_pre_) up to angles of ~60° (Figure 2E). When the distribution of turning angles (Δθ) was normalized to the occurrence of fish-target angles, turning angles were distributed uniformly within this range (Figure 2F). Apparently, the fish attempts to minimize the angle between its body axis and the location of the prey within one swim, and it does so with a precision of |ϕ_post_| = 8.06° ± 1.7° when the fish-target angle is in the range 0° < ϕ_pre_ < 60° (Figure 2G). In general, we observed more undershoot in turning (64%, 69 out of 107 swims) than overshoot (36%, 38 out of 107 swims). Note that large-angle turns preferentially occurred at early stages of the sequence compared to small-angle turns (see color-coded order of swims in Figures 2D,G,H). Noteworthy, the number of tail beat cycles per swim was not significantly different (One-Way ANOVA, *p* = 0.32, *n* = 107) for large and small angle turns (mean number of tail beat cycles per swim for 1st, 2nd, 3rd, and 4th swim was 2.92 ± 0.08, 3.02 ± 0.1, 2.88 ± 0.09, and 2.74 ± 0.1, respectively). We observed that the duration of the individual swim bout decreased slightly during the progression of the sequence: the mean duration of 1st, 2nd, 3rd, and 4th swim was 141 ± 2.8, 136 ± 4.5, 124 ± 3.4, and 116 ± 4.9 ms, respectively. This temporal compression is explained by the observation that the duration of the first tail beat cycle varied with the change in orientation (Δθ) in a graded fashion (Figure 2H), and larger changes predominantly occurred during the first and second swim.

This suggests that the larva controls the degree of turning smoothly by an asymmetric bend component of the tail, which prolongs the duration of the first tail beat cycle. More generally, the prey capture sequence appears to be composed of an elementary swim pattern, or motor primitive (Grillner, 1981; Bizzi et al., 2000), whose angular bias is modulated on a graded scale by the fish-target angle ϕ_pre_ preceding the swim by some 10s of milliseconds. The beginning of a sequence is effectively triggered by small objects subtending <5° visual angle moving at speeds of ~20°/s (Figures 1G,H). This may represent a trigger feature for prey capture behavior in larval zebrafish. Since in 30 sequences, fish engaged in prey capture at initial fish-target angles of 20° < ϕ_pre_ < 105°, i.e., when the prey was invisible to the contralateral eye, the sequence seems to be preferentially initiated in a monocular sub-circuit of the visuo motor pathway.

### Low frequency content of individual swim bouts predicts the change in orientation

**Figure 3:**
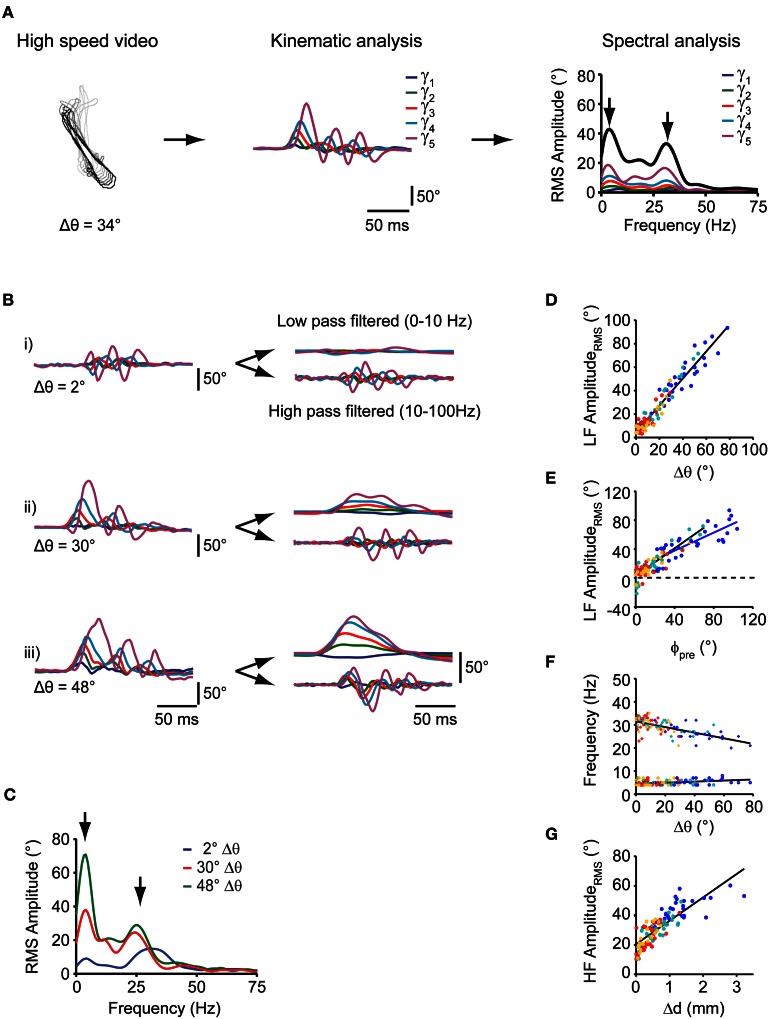
**Spectral analysis of individual swim bouts of the freely moving larva during prey capture. (A)** Schematic representation of spectral analysis of single swim bouts. Left: fish contours of a swim bout. Center: time course of tail angles (γ_1_, …, γ_5_) obtained from automated tail angle measurement. Right: Fourier transform of each of the tail angle traces (γ_1_, …, γ_5_) results in individual RMS amplitude spectra shown in the corresponding color. Sum of these individual spectra is shown as bold black curve. Note two peaks in the spectra (arrows). **(B)** Left: time course of tail angles (γ_1_, …, γ_5_) for the three swim examples shown in Figure 2C. Right: time course of tail angles after low- and high-band-pass filtering. **(C)** Summed RMS amplitude spectra for the three swim bouts shown in **(B)**. Note two peaks in the amplitude spectra (arrows) at frequencies similar to those in (**A**, right panel). The peak at lower frequencies (~4 Hz) scales with change in orientation (Δθ). **(D)** Scatter plot of the low-frequency (LF) peak amplitude from spectral analysis vs. change in orientation (Δθ). Colors indicate swim bout number (as in Figure 2). **(E)** Scatter plot of the low-frequency peak amplitude from spectral analysis vs. fish-target angle (ϕ_pre_) preceding the swim. Solid lines: straight line fits to the data pairs from first swims (blue) and subsequent swims (black) during a sequence. Note shallower slope for first swims. Same color code as in **(D)**. **(F)** Scatter plot of the location of the high frequency peak (crosses) and low frequency peak (circles) vs. the change in orientation after each swim (Δθ). Lines are straight line fits to the data. Same color code as in **(D)**. **(G)** Scatter plot of high-frequency (HF) peak amplitude vs. change in fish-target distance (Δd) after the swim bout. Same color code as in **(D)**.

Next, we sought to predict the change in orientation (Δθ) of this elementary swim pattern from measurements of tail angle kinematics. The waveforms of the tail angles γ_1_, …, γ_5_ were extracted from the recorded swim patterns and were analyzed to further characterize individual motor patterns. We calculated the spectral composition of swim bouts during prey capture using discrete Fourier analysis. Single-sided amplitude spectra were computed for each tail segment waveform individually and subsequently summed (Figure 3A, and Materials and Methods). This analysis was applied to all swim bouts comprising varying degrees of turning (Figure 3B,i–iii, left panels, and 3C).

Notably, swim bouts during prey capture exhibited a peak in the sum spectrum at a low frequency between 3 and 5 Hz (Figure 3C, left arrow), whose peak amplitude (“LF amplitude_rms_”) showed a strong correlation with the change in orientation Δθ (*r*_Pearson_ = 0.97, *p* < 10^−10^; Figure 3D) and with the fish-target angle ϕ_pre_ (*r*_Pearson_ = 0.9, *p* < 10^−10^; Figure 3E). Furthermore, the location of the LF peak was constant and did not vary with the change in orientation Δ θ (slope = 0.02, *r*_pearson_ = 0.38, *p* = 0.03, Figure 3F, circles). The peak at 3–5 Hz was also present in the spectra of the individual tail segment waveforms (Figure 3A, right panel). The slope of the linear fit between the LF amplitude_rms_ and the fish-target angle (ϕ_pre_) was lower for first swims (slope = 0.62) than for subsequent swims (slope = 0.95; Figure 3E). This can be attributed to the observation that the fraction of turns that undershot the target was larger for first swims, when the fish-target angles ϕ_pre_ tend to be large (Figure 2G). Furthermore, the graded relationship between the low frequency component and the turning angle becomes apparent when the tail segment waveforms are filtered at low and high band-pass settings. While the high-frequency components of the tail segment angles oscillate symmetrically around 0, the low frequency components reflect the amount of turning on a continuous scale (Figure 3B, right panels).

Second, we found that all swims had a pronounced peak at higher frequencies in the sum spectrum at 28.2 ± 0.56 Hz (range: 20–35 Hz, *n* = 107 swims; Figure 3C, right arrow), which corresponds to the time-averaged tail beat frequency during a swim. The location of the high frequency peak varied mildly with the change in orientation Δθ during the swim (slope = −0.11, *r*_Pearson_ = 0.64, *p* < 10^−10^, Figure 3F; crosses), but never exceeded 35 Hz. This is much lower than the maximal tail-beat frequency larvae at this stage can employ during other behaviors, e.g., escape [up to ~80 Hz; (McLean et al., 2008)]. We also noted that the larva approached the prey within a swim by variable amounts, ranging between 0 and 3 mm, with larger distances covered at earlier stages of the sequence (Figure 3G). While the tail beat frequency, the number of cycles per swim and the swim duration were relatively constant across all swims, we observed that the HF amplitude_rms_ was strongly correlated with the distance traveled during a swim (*r*_Pearson_ = 0.82, *p* < 10^−10^; Figure 3G). This indicates that the fish regulates its forward drive by amplitude modulation during prey capture behavior. Taken together, the relatively constant peaks in the frequency spectrum, the smooth distribution of changes in orientation covering a large range of angles, and the constant number of tail beat cycles suggests that an elementary motor pattern is employed at all stages of the prey capture sequence (other than the capture swim).

### Visually evoked swims in restrained larvae mimic motor patterns of the prey capture sequence

**Figure 4:**
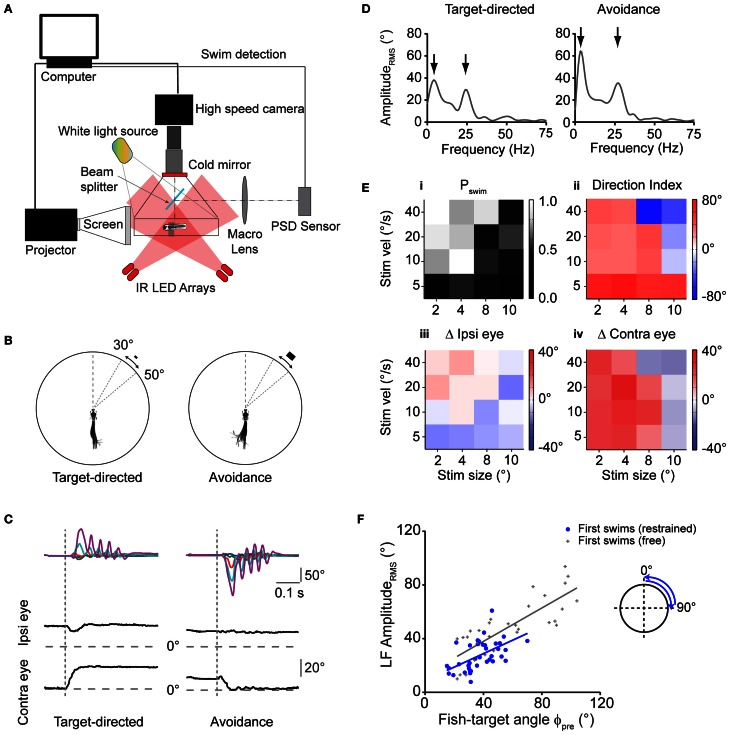
**Virtual prey-like stimuli evoke swims similar to motor patterns during prey capture. (A)** Illustration depicting the setup used to record swim bouts in response to visual stimuli presented to minimally restrained larvae. A position-sensitive device (PSD, right) is used to detect swims and update the visual stimulus at high speed. **(B)** Left: swim bout toward a rectangular stimulus (width × height: 2 × 1°), moving at 20°/s peripherally between 30 and 50°. Right: swim bout toward the opposite direction of a rectangular stimulus (8 × 4°) moving at 40°/s. Every 10th frame of the high speed movies during the swim bout is overlaid. Angular dimensions drawn to scale. Fish and chamber dimensions not drawn to scale. **(C)** Time course of tail angles and ipsilateral and contralateral eye angles for the examples shown in **(B)** obtained using automated image analysis. Same color code as in Figure 3A. Note: high spatial resolution during imaging of restrained larvae allowed automated eye angle analysis. **(D)** Summed RMS amplitude spectra obtained from traces in **(C)** for the target-directed (left) and avoidance swim bout (right). Note two peaks in the spectra (arrows), similar to spectra measured in freely moving larvae during prey capture. **(E)** Summary of motor output in response to 16 different combinations of size and velocity of a moving stimulus. Panel **(i)**: probability of observing a swim bout during a 60 s interval of stimulus presentation. Panel **(ii)**: direction index calculated from LF peaks in the amplitude spectrum, showing target-directed, and avoidance turns (red and blue squares, respectively). Panel **(iii)**: change in position of ipsilateral eye. Positive values indicate rotation to more nasal position. Panel **(iv)**: same as panel **(iii)**, but for contralateral eye. Positive values indicate rotation to more nasal position. In panels **(ii–iv)**, colors indicate mean values (*n* = 6–12 trials for each stimulus parameter pair). Note: for each panel, the value of the target size denotes the width of the stimulus and width:height ratio is always 2:1. **(F)** Scatter plot of the LF peak amplitude_rms_ from spectral analysis vs. fish-target angle (ϕ_pre_) immediately preceding the swim. A trial consisted of the stimulus moving from center to periphery (rostro-caudal) or periphery to center (caudo-rostral), where it disappeared. Stimulus size and velocity 2° and 20°/s, respectively. Blue solid line: straight line fit. Also shown are data pairs (LF peak amplitude_rms_; ϕ_pre_) and straight line fit from first swims in freely moving larvae (gray symbols; same data as in Figure 3E).

Detailed analysis of prey capture behavior showed that freely moving larvae use an elementary, directionally graded motor pattern that is chained into a sequence in order to approach and catch prey. Next, we tested whether this swim pattern can be evoked in minimally restrained larvae using artificial stimuli in a virtual environment. To create a virtual reality, we positioned the larva with the head in the center of a quartz glass chamber, held in place by a thin collar of agarose at the level of the ear that allowed the larva to move its eyes and tail freely. Using a DLP projector, computer-generated stimuli were projected onto a screen that subtended a horizontal visual angle of ~110° centered around the heading direction of the fish. Visual targets consisted of small white rectangles of various sizes moving with different velocities against a dark structured background. The motor behavior of the fish was recorded using infrared darkfield illumination and high speed video recordings (Figure 4A).

First, we sought to identify stimulus conditions that could serve as a trigger to initiate the first swim of a prey capture sequence. We found that small moving rectangles readily evoked short directed swim patterns similar to those observed in the freely moving larva during prey capture (Figure 4B). Specifically, we observed both directed swims toward the moving target with small target size as well as avoidance swims directed away from it when the size and velocity of the stimulus were increased, consistent with earlier results in freely moving larvae (Bianco et al., 2011). Notably, target-directed swims were accompanied by contralateral eye convergence, which was a hallmark of first swims in our recordings of prey capture sequences. By contrast, we observed contralateral eye divergence during avoidance swims (Figure 4C, left vs. right panel).

Using automatic image analysis, we quantified tail and eye angles in the high-speed video recordings to determine the dependence of motor output on target size and velocity (Figure 4C). Summed, single-sided amplitude spectra were computed from tail angles γ_1_, …, γ_5_ as described above, which showed a low frequency peak at 3.63 ± 0.07 Hz, and a high frequency peak at 26.3 ± 0.26 Hz (*n* = 75 swims), similar to motor patterns during free swimming, for both target-directed and avoidance swims (Figure 4D, left vs. right). A direction index (DI) was assigned to each swim, which was the low frequency peak of the amplitude spectrum, multiplied by the sign of the average tail angle integral, so that target-directed and avoidance swims had a positive and negative DI, respectively (Materials and Methods).

During free prey capture, the fish encounters prey objects of different angular sizes and velocities at early and late stages of the sequence (Figures 1G,H). We tested 16 pairs of target size and velocity that can occur under realistic conditions of the prey capture sequence (Figure 4E). Stimuli were most effective in triggering a first swim in a 60 s stimulus trial when they were small and moved at moderate to high velocity (Figure 4E,i, upper left corner), whereas the probability of evoking a swim declined with larger target sizes and lower velocities. Target-directed swims preferentially occurred when the stimulus was small (positive DI; Figure 4E,ii, left half), and were accompanied by convergence of the contralateral eye (Figure 4E,iv), which supports the notion that first swims of a prey capture sequence were evoked. By contrast, avoidance swims were evoked preferentially by larger targets moving at high velocities (negative DI; Figure 4E,ii, upper right corner) and were accompanied by contralateral eye divergence (Figure 4E,iv). In both cases the ipsilateral eye exhibited smaller positional changes (Figure 4E,iii).

Next, in order to test whether the asymmetric bend component of the tail varied with the fish-target angle in a graded way, we used targets that moved unilaterally in a wider range of [0°, 90°] in one half of the visual field (Figure 4F, inset). From single-sided amplitude spectra (not shown), we observed that the spectral amplitude at 4 Hz covaried with the fish-target angle ϕ_pre_. The slope of a linear fit was 0.5 and the y-intercept was 9.8° (Figure 4F, blue straight line), similar to the slope and y-intercept of the same relationship during first swims in freely moving larvae (Figure 4F, gray straight line, slope = 0.62, y-intercept = 13.1°). Slope and y-intercept were not statistically different in the two conditions (ANCOVA, *p* = 0.45 and *p* = 0.62 for slope and y-intercept, respectively). This suggests that the fish performed graded, target-directed swims similar to first swims that define the start of prey capture sequences in freely moving animals. On visual inspection, the LF amplitude_rms_ tended to be somewhat smaller in the restrained case, which reflects an overall reduction of tail segment angles during intended turning, and is probably caused by a larger drag force on the tail when the larva's head is held in a fixed position. Finally, the number of tail beat cycles in these swims was 5.82 ± 0.18 and the mean swim duration was 287 ± 9 ms (*n* = 39 swims). This increase in swim duration in the restrained larva is consistent with a prolonged duration of swim bouts observed in the fictive swim preparation (Buss and Drapeau, 2001) and may be caused by the altered sensory (e.g., visual, vestibular, somatosensory) feedback when the larva is partially restrained in agarose. However, it should be noted that the location of the low and high frequency components in the swim spectra of restrained larvae (Figure 4D) were very similar to those in the freely moving case, suggesting that the motor patterns observed in the restrained experiments were comparable and also elementary in nature.

### Virtual prey capture in a closed-loop projection system

**Figure 5:**
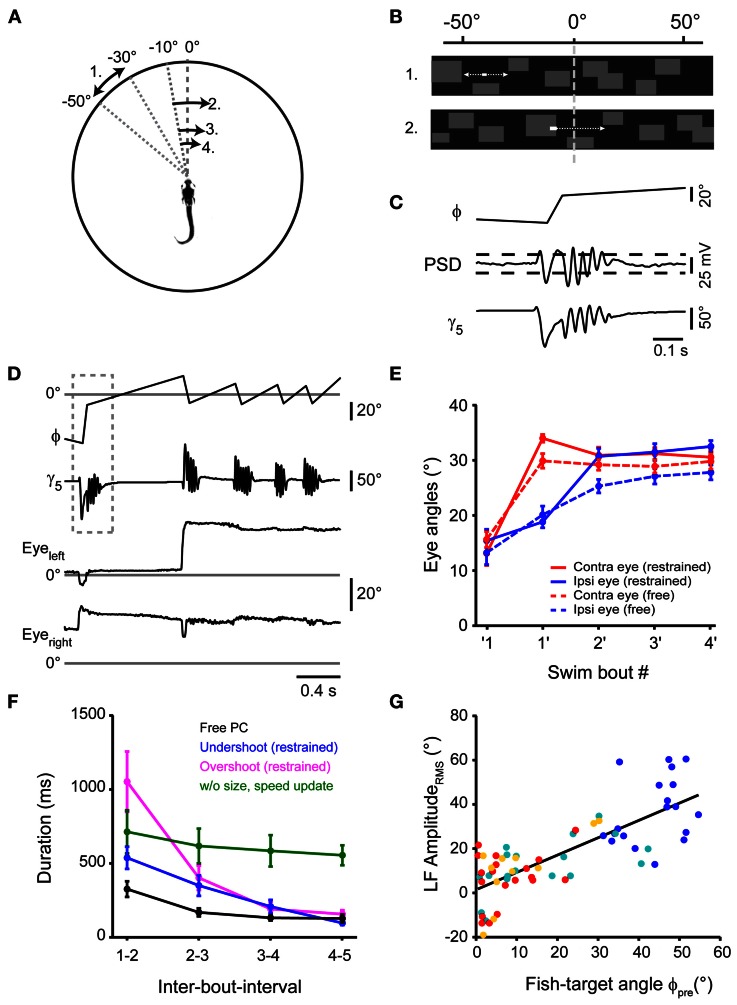
**Virtual prey capture behavior in a closed-loop system. (A)** Illustration depicting the stimulus update paradigm used to evoke prey capture-like swim sequences in minimally restrained larvae. Numbers indicate order of appearance of moving stimulus, arrows indicate direction. Here, following detection of a swim, the stimulus was translated to −10°, from where it moved across the midline into the contralateral visual hemifield. Target size and velocity were increased following each swim, emulating an approach of the larva toward the target. This is illustrated by reducing the distance of arrows in the diagram. **(B)** Representation of the stimulus from the perspective of the larva. Initially, a rectangular target moves in the periphery against a background of low spatial frequency content (upper panel). At the onset of a swim bout, target and background translate smoothly toward the visual field center (within ±10°, update velocity ~400°/s), emulating a change in orientation of the fish toward the target. Subsequently, the target continues to move against the background (lower panel). **(C)** Detection of swim bouts. Bottom: time course of caudal tail angle γ_5_, during a target-directed swim bout. Center: time course of PSD voltage during the swim. Top: fish-target angle (ϕ) before, during, and after the swim bout. Target and background rotation were triggered in real-time by threshold-crossing of the PSD signal (indicated by dashed horizontal lines). **(D)** A swim sequence resembling prey capture sequences in freely moving larvae, recorded in a minimally restrained larva. Top: time course of fish-target angle ϕ. Second from top: caudal tail angle γ_5_. Dashed box indicates temporal window shown in **(C)** on an expanded scale. Bottom two traces: left and right eye angle traces. **(E)** Comparison of ipsilateral eye angles (15.4 ± 2.1° before first swim; 32.5 ± 1.1° after 4th swim) and contralateral eye angles (13.1 ± 2.2° before first swim; 30.6 ± 1.3° after 4th swim) during sequences in restrained larvae (solid line; *n* = 19 sequences) to eye angles in freely moving larvae (broken line; *n* = 30 sequences). **(F)** Comparison of inter-bout-intervals (IBIs) for different stimulus conditions. Black line: IBIs during prey capture sequences in freely moving larvae (*n* = 30 sequences). Blue line: IBIs during sequences of restrained larvae, where target is translated to −10° during the first swim, representing undershoot (*n* = 11 sequences). Magenta line: IBIs during sequences of restrained larvae, where target is translated to +10° during the first swim, representing overshoot (*n* = 8 sequences). Green line: IBIs during sequences of restrained larvae, where target is translated to −10° during the first swim, representing undershoot, but without increases in stimulus size and velocity throughout the sequence (*n* = 6 sequences). **(G)** Scatter plot of the LF peak amplitude from spectral analysis vs. fish-target angle (ϕ_pre_) immediately preceding the swim (*n* = 19 sequences). Same color code as in Figure 2.

Restrained fish performed first swims in response to small projected targets moving at a realistic speed. Next, we intended to close the loop of the virtual reality system to generate an update of the visual world similar to what the fish would experience when orienting during natural prey capture (Figures 5A,B). During orienting swims, the change in orientation is accomplished within 1–2 cycles of the tail beat, i.e., in <100 ms (compare Figure 2B, inset). Therefore, to implement an update of the visual world with sufficiently small delays, we equipped the virtual reality setup with a fast, position-sensitive device (PSD) in parallel to the high-speed camera (Figure 4A, right arm). Thus, deviations of the tail position during swimming were detected in real time. Threshold-crossing of the PSD signal was used to trigger an update of the visual stimulus in <60 milliseconds following the onset of a swim bout (Figures 5B,C). The update consisted of a translation of the visual stimulus and background within 80 ms to a position within ±10° from the central position of the screen, from where the stimulus continued to move toward the periphery against a stationary background (Figures 5B,C). The fish often responded to the updated stimulus with a second target-directed swim, which triggered a second stimulus update to ±10° from the center. Typically, three or more swim bouts could be evoked this way in rapid succession, very much resembling prey capture sequences in freely moving larvae (Figure 5D; Movies S3, S4). Importantly, these evoked sequences were accompanied by a two-step eye convergence pattern during the first and second swim (Figure 5E), similar to the natural pattern during free hunting (Figure 1D). In order to present realistic stimulus cues, we updated the stimulus size and velocity according to the measured values observed during free prey capture behavior, which could serve as distance cues to the animal. Notably, the IBIs between evoked swims decreased rapidly during the virtual prey capture sequence, similar to the rapid shortening of IBIs during free prey capture sequences (Figure 5F). It should be noted that IBIs also depended on the update position after the first swim. When an undershoot in turning was simulated by updating the stimulus to a position of −10° from where it moved across the midline into the hemifield of the contralateral eye (Figure 5F, blue line), IBIs were systematically faster than when an overshoot in turning was simulated by updating the stimulus beyond the midline to +10° after the detection of the first swim (Figure 5F, magenta line). This indicates that the fish is sensitive to small changes in update location, with a significant slowing of reaction times when the fish virtually overshoots the target. Furthermore, when the size and velocity of the updated moving target were kept constant during a virtual prey capture sequence, resembling a distant moving prey without approach, the fish still performed multiple target-directed swims, albeit with negligible reduction in IBIs (Figure 5F, green line). Together, these findings suggest that once the prey capture sequence has started, the stepwise increases of stimulus size and velocity serve as features of a visual feedback signal that may accelerate the programming and execution of subsequent swim bouts.

Finally, we also analyzed the relation between the DI of each swim and the instantaneous fish-target angle ϕ_pre_ before the swim (Figure 5G). The DI was correlated with ϕ_pre_ for early and late swims in the evoked swim sequence (*r*_Pearson_ = 0.77, *p* < 10^−10^), similar to sequences in freely moving larvae. After 4–5 swims, the fish typically stopped responding or performed a long-duration (>500 ms) struggle swim. This suggests that the visual feedback late in the sequence had become inappropriate, which led to a departure from the normal behavioral trajectory. In conclusion, these data show that the classical sequential prey capture behavior can be evoked in a closed-loop virtual environment with fast visual feedback and realistic update of visual target properties.

### Timing of sequenced swims depends on target update location

**Figure 6:**
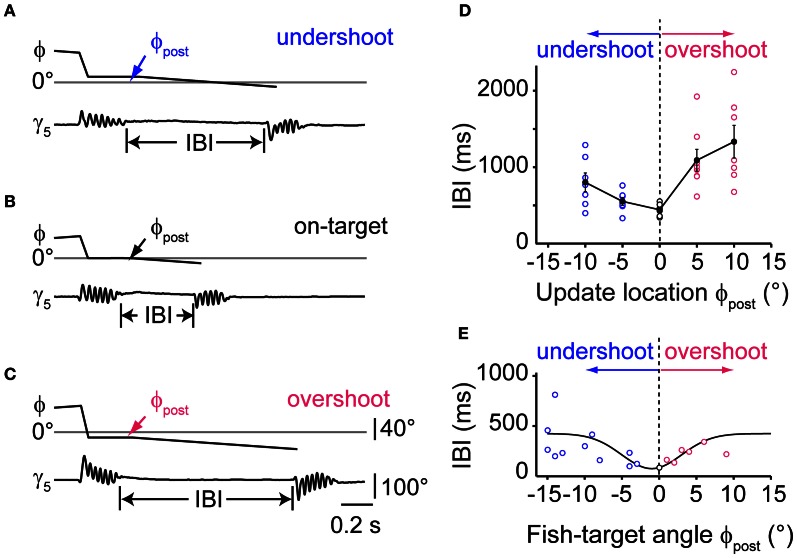
**Timing of sequenced swim bouts depends on updated stimulus location. (A)** Time course of fish-target angle ϕ and caudal tail angle γ_5_ during a pair of swim bouts evoked by a moving target in a restrained larva. First swim was directed toward a stimulus moving in the periphery (35–55°). Onset of the first swim triggered a translation of stimulus/background stopping short 10° from the midline, simulating an undershoot in turning. The end of the first swim triggered stimulus motion at constant velocity toward the contralateral hemifield, evoking a second, target-directed swim. Inter-bout-interval (IBI) indicated by vertical lines. **(B)** Same as in panel **(A)**, but with the stimulus/background translated to the center of the visual field (0°) during the first swim, simulating exact alignment of the larva with the location of prey (“on-target”). Note that the IBI is considerably shorter. **(C)** Same as in panel **(A)**, but with the stimulus/background translated beyond the center of the visual field by 10° during the first swim, simulating an overshoot in turning. **(D)** Dependence of IBIs on the update location of the stimulus during the first swim bout. Trials with initial stimulus position on the left or right side were interspersed and pooled. Negative values of update location represent an undershoot; positive values an overshoot during the first swim. Data from *n* = 6 fish. **(E)** Data from recordings of freely moving larvae performing prey capture sequences. Scatter plot of IBIs between first and second swim bout in which the fish-target angle ϕ_post_ (measured at the end of the first swim) varied between ±15°. Negative values correspond to an undershoot, positive values to an overshoot in turning. Note the minimum in IBIs for small turning error near ϕ_post_ = 0°. Solid line is a Gaussian fit curve.

To further delineate the influence of single stimulus properties on the IBIs of second swims, we systematically varied the update location after the first swim in a narrow range around the center of the visual field while keeping update size and velocity constant, (Figures 6A–C). The PSD-detected onset of a first swim triggered a translation of the visual target and background to near center positions. The target was maintained at this pre-specified position until the end of the first swim (as estimated from the PSD signal in real time), after which it continued to move into the contralateral field. We observed that small changes in the updated stimulus location had a significant effect on the IBI (Figure 6D, One-Way ANOVA for means, *p* = 0.0003). A minimal IBI of 442 ± 33 ms (*n* = 6) was observed when the target was updated to 0°, simulating a perfectly aligned orientation at the end of the first swim. Small deviations in update position ranging between ±10° resulted in significantly longer IBIs (two sample *t*-test, *p* = 0.0055 and *p* = 0.0015 for 10° undershoot and overshoot, respectively). Based on this observation, we analyzed whether the IBIs between first and second swims during prey capture in freely moving larvae showed a similar dependence on target position after the first swim. In 18 sequences, in which the fish-target angle after the first swim was between −15° and +15°, minimal IBIs were observed for post-swim fish-target angles near 0°, in good agreement with the data from the virtual reality experiment (Figure 6E). This suggests that the larva's visual system is remarkably sensitive to the perceived error in turning and can trigger second swims more rapidly when the target is at a central position. Furthermore, the deviation of the target from central position may generate a corrective signal that is used for computing asymmetric turning bias for a second swim, which may require additional processing time.

### Timing of sequenced swims depends on update delays

**Figure 7:**
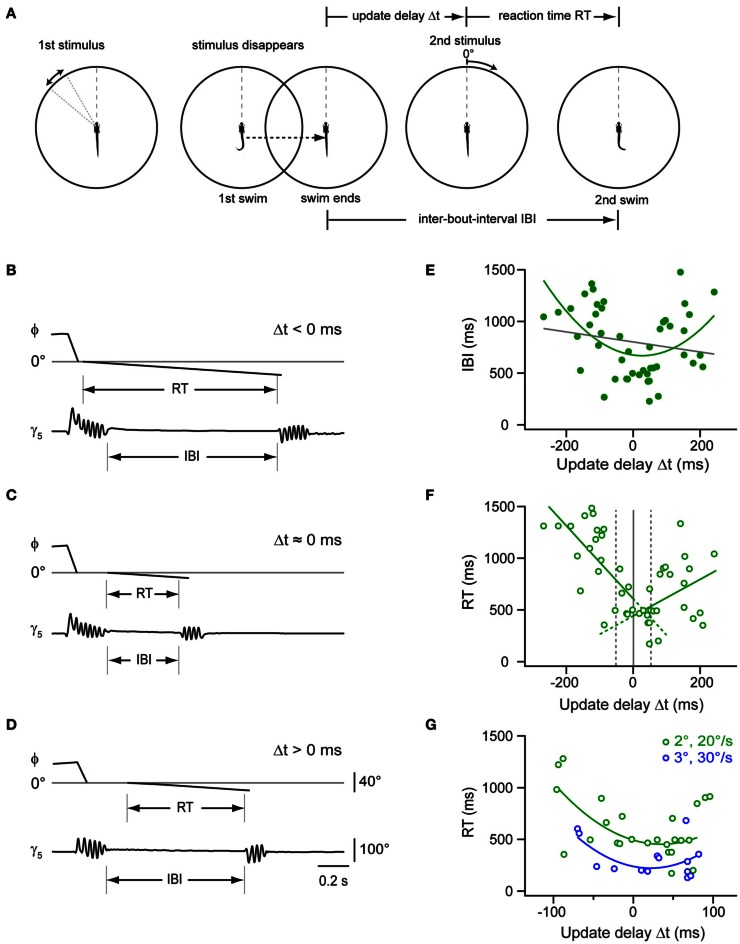
**Impact of stimulus timing on inter-bout-intervals and reaction times in a two-step stimulus paradigm. (A)** Schematic of a two-step stimulus paradigm with variable delay. First stimulus is a target moving in the periphery (35–55°; left panel), which eventually triggers a target-directed swim (second panel). Stimulus and background translate to center (0°), and the stimulus disappears. After a variable delay, the target reappears at the center and moves toward the periphery at constant size and speed (2°; 20°/s; 3rd panel), until the larva performs a second directed swim, which ends the trial (right panel). Update delays (Δt) and inter-bout-intervals (IBI) are measured relative to the end of the first swim, reaction time (RT) is measured from onset of second stimulus. **(B)** Time course of fish-target angle ϕ and caudal tail angle γ_5_ during paired swim bouts evoked by the two-step stimulus paradigm. The second stimulus appeared before the end of the first swim, corresponding to a Δt < 0 ms. Note long IBI. **(C)** Same as in **(B)**, but with second stimulus appearing near end of first swim (Δt ≈0 ms). Note short IBI. **(D)** Same as in **(B)**, but with second stimulus appearing after end of first swim (Δt > 0 ms). Note longer IBI. Scale bars apply to panel **(B–D)**. **(E)** Scatter plot of IBIs vs. update delay (Δt). Gray line: straight line fit (r_Pearson_ = 0.18). Green curve: second order polynomial fit to the data (*r* = 0.46). Note minimum near Δt ≈ 0 ms (*n* = 47 trials from 13 fish). **(F)** Scatter plot of reaction times (RT) vs. update delays (Δt). Green lines: straight line fits to data points with negative and positive update delays (Δt), respectively (*n* = 47 trials from 13 fish). Broken gray lines represent three different delay groups i.e., Δt < -50 ms, -50 ms < Δt < 50ms, and Δt > 50 ms. **(G)** Scatter plot of RT values vs. update delays (Δt) on an expanded time scale (–100 to 100 ms). Reaction times are shorter when the second stimulus is larger and faster (3°; 30°/s; *n* = 14 trials from 5 fish; blue symbols) than under control conditions [2°; 20°/s; green symbols, same as in panel **(F)**]. Blue and green curves are second order polynomial fits to data points measured under the two conditions, respectively.

The previous experiment showed that the larva proceeds rapidly in the motor sequence when the first swim brings the target into a central position. This could mean that a window of expectation is opened, which enables the larva to perform accelerated swims if expected and true target position overlap. To measure the time course of this window, we programmed the stimulus to appear at the optimal target position of 0°, but varied the update to occur with a delay between 100 and 500 ms after the onset of the first swim (Figures 7A–D). This stimulus paradigm resulted in sweeps in which the stimulus reappeared and moved either shortly before or after the end of the swim, resulting in negative or positive delay values (Δt), respectively. Notably, we observed that the IBIs were minimal when the stimulus reappeared around the end of the swim (Figures 7C,E). By contrast, when the stimulus returned earlier or later, longer IBIs were observed (Figures 7B,D,E). We also determined the reaction time, which exhibited a minimum for delay values around the end of the swim as well (Figure 7F). Notably, when comparing trials in which Δt < −50 ms, the reaction time was significantly longer than in those trials where the stimulus appeared at the end of the swim (within Δt ±50 ms, two-sample *t*-test, *p* < 10^−5^). This suggests that the larva is less sensitive to visual feedback while swimming. Similarly, when the second stimulus was presented only ~100 ms after the end of the first swim (Figures 7D–F), reaction times increased again, indicating that the window of expected stimulus processing closes shortly after the swim.

We also tested whether increasing the size and velocity of the second stimulus could further reduce this minimal reaction time. With a second stimulus of larger size and velocity (3°, 30°/s), we observed second responses with a reaction time of 318 ± 47 ms (*n* = 14 trials; Figure 7G). This was significantly faster than the reaction time observed with second stimuli at unchanged size and velocity (622 ± 60 ms; two sample *t*-test, *p* = 0.0006, averaged for trials with Δt within ±100 ms). The duration of the first swims for the two differing update conditions was not significantly different (two sample *t*-test, *p* = 0.21). Hence, the shortening in reaction time can be attributed to the change in size and velocity of the stimulus. In conclusion, larval zebrafish showed variable IBIs and reaction times between the first and second swim of a virtual prey capture sequence, with a minimal IBI when the stimulus reappeared after a simulated turn near 0° at the end of the first swim. This lends support to the notion that during hunting, the larva uses visual feedback in-between swims to compare the observed location with an expected target position, which enables the larvae to perform more rapidly if predicted and observed target positions agree in space and time.

## Discussion

Here, we present a quantitative description of prey capture behavior with respect to several visual stimulus parameters and behavioral output. Quantitative analysis of fish-target angles preceding a swim bout and change in orientation after the swim bout showed that there is a graded relationship between visual input and motor output (Figure 2). We further quantified the kinematics of each of these bouts using spectral analysis to show that a graded continuum of seemingly different swim patterns can be produced by modulating one elementary motor pattern. In order to test the boundary conditions for input space under which prey capture sequences are observed, we developed a closed-loop visual environment that is feedback driven by the larval motor output. By deriving a combination of stimulus parameters from naturally occurring prey capture, we elicited behavioral sequences in restrained larvae using virtual prey stimuli. Such visually guided behavioral sequences were very similar to prey capture in freely moving larvae with respect to spectral components of individual swim bouts as well as in the monotonically decreasing inter-bout intervals. By taking advantage of the precise control over stimulus parameters and visual feedback, we manipulated the timing, location, size, and velocity of the stimulus following the detection of a swim bout. This allowed us to probe how visual feedback could influence the timing between two swims.

### Elementary motor pattern during prey capture sequences

The discrete swim bouts during prey capture sequences (except for the final capture swim) appear to be versions of an elementary motor pattern, modulated on a graded scale by an asymmetric turning component. The notion that only one basic motor pattern is modulated and chained into a sequence is supported by our observation that early and late swim bouts in the sequence employed a rather constant tail beat frequency of ~30 Hz, which did not appreciably vary with change in orientation. Also, swim bouts at all turning angles exhibited a constant number of cycles per swim. Finally, we observed that this basic swim pattern enabled the larva to turn on a graded scale within a range between 0° and ~60°, and that turning angles were distributed continuously within this range (Figures 2D–F). This may represent a departure from the notion that larval zebrafish use different classes of motor patterns during prey capture, such as “slow swims” for forward swimming and “J-turns” for orienting swims (McElligott and O'Malley, 2005; Bianco et al., 2011). Instead, the motor pattern may consist of a basic burst-like tail beat component, symmetrically oscillating at ~30 Hz and terminated after ~150 ms. Onto this basic pattern, a slower, asymmetric turning component may be superimposed, which biases the bending of the tail toward the desired side of turning. The turning component appears to be freely adjustable within a large angular range, and controlled by the angular position of the prey (Figures 2D and 3E).

What could be the organization of the neural commands underlying such a directionally graded, but temporally rather uniform motor pattern during prey capture? Based on the stereotypical dynamics of the basic motor pattern, we hypothesize that a symmetric command component (S) from a subset of descending reticulospinal (RS) neurons serves as a trigger to initiate a swim bout, whose frequency is set by the characteristic frequency of pattern generator modules in the spinal cord (Grillner et al., 1991; Wiggin et al., 2012). This signal may be symmetrically distributed about the midline of the RS system; currently, it is unclear which RS cells in the zebrafish hindbrain may be involved in carrying such a bilateral signal. By contrast, a second command component (A) may be asymmetrically distributed in descending RS neurons, which could evoke more tonic muscle recruitment on the turning side to drive unilateral tail displacement. It is unclear whether this asymmetric component (A) is carried by the same descending RS neurons that also mediate the swim initiation command (S) or by different RS neurons. It has been shown that activity in specific RS cell types, such as MiV cells, correlates with change in orientation during the optomotor response (Orger et al., 2008). This, together with the large number of descending RS neurons suggests that the S-component and the A-component could be assigned to different RS cell types also during prey capture. Furthermore, it is not known whether this elementary swim pattern is actively terminated or whether its duration reflects the intrinsic decay of activity in a damped oscillator circuit (Wyart et al., 2009). We observed that swim bouts in restrained larvae were longer than those observed during freely moving behavior. This suggests that the duration of elementary swim patterns may be controlled by the duration of descending command signals, which has been observed for longer swim episodes in lamprey (Deliagina et al., 2000). The duration of such descending “gate” signals that drive swimming, or alternatively, the timing of a descending “stop” signal that could terminate a swim (Roberts et al., 2008), may in turn be controlled by visual or non-visual sensory feedback due to the self-motion during the swim, which is lacking in the restrained larva, and could therefore explain the difference in swim duration.

Looking upstream, we may ask what neural mechanisms may generate the trigger/gating component (S) and the position component (A) of the command signals for this elementary motor pattern. Earlier loss-of-function experiments using laser ablation have shown that the tectum is essential for directed turning and successful prey capture in larval zebrafish (Gahtan et al., 2005). Furthermore, bulk-loading and stochastic single-cell labeling techniques demonstrated the presence of direct ipsi- and contralateral projections from the tectum to the ventral neuropil of the hindbrain, where descending RS neurons could receive direct synaptic input via their ventral dendrites (Metcalfe et al., 1986; Sato et al., 2007; Robles et al., 2011). On a functional level, Ca^2+^ imaging in the tectum demonstrated a retinotopic map of visual space onto the tectal cell population both for artificial stimuli and for natural prey objects (Niell and Smith, 2005; Muto et al., 2013). Taken together, these findings corroborate the notion that the angular position of the targeted prey is encoded in the location of activity in the retinorecipient layers of the tectal neuropil. Tectal location of activity, in turn, is thought to be transformed into a rate-coded motor command signal at the level of brainstem command neurons (Scudder et al., 2002). Experiments using electrical stimulation of the tectum in goldfish showed that stimulus location correlates with the evoked tail bend amplitude, consistent with this model (Herrero et al., 1998). It has been suggested that projection neurons from rostral and caudal regions of the tectum may form synapses onto command neurons in the reticular formation with increasing synaptic weights, which could explain this transformation of location of activity into firing rate (Moschovakis et al., 1998; Groh, 2001). To test this model in zebrafish, it will be important to investigate whether projection neurons from rostral and caudal regions of the tectum drive activity in the same set of RS neurons with different synaptic efficacies.

### Multistep sequences and visual feedback

In freely moving larvae, we observed a characteristic two-step eye convergence pattern during the first two directed swims toward the prey. This two-step convergence pattern was also observed during closed-loop visual stimulation in our virtual reality prey capture assay. While this is generally consistent with the recent proposal that eye convergence is associated with the first swim bout of the capture sequence, we did not observe that convergence movements rotate both eyes symmetrically to the nasal limit of the oculomotor range within a single swim (Bianco et al., 2011). Rather, the ipsilateral eye converged only partially during the first swim, with weaker convergence typically occurring during large angle turns. This suggests that convergence of the two eyes can be controlled independently. During the first swim, the ipsilateral eye position may depend on the combination of a divergence signal, which scales with the asymmetric turning command component, and a competing convergence signal, which drives the eye toward its nasal limit. A possible reason for the different observations in eye convergence may lie in the different experimental conditions, e.g. the high density of paramecia used in the earlier assay (>200 paramecia per dish, Bianco et al., 2011), compared to a single paramecium in our assay. This can also explain the different average distances of targeted prey at the onset of the sequence (3.8 mm in our assay vs. 1.55 mm in the earlier study). In a high density assay, the larva is likely to simultaneously encounter multiple prey objects within its visual range and may tend to perform low-angle swims to the target nearest to its heading direction.

The prominent burst-and-pause swim pattern employed by the larva during prey capture falls into the class of intermittent locomotor behaviors. This sample/move strategy is employed during pursuit behavior by various species such as flies (Boeddeker and Egelhaaf, 2005), lizards (Avery et al., 1987), and toads (Lock and Collett, 1979; Ewert, 1987) and can be compared to saccadic eye movements during visual search in the primate (Land, 1992, 1999; Schall and Thompson, 1999). It has been generally reasoned that this mode of locomotion may be ecologically advantageous to enable short-lasting states of intermittent rest, during which prey or predatory objects may be detected more reliably due to the lack of sensory input generated by self-motion [reviewed in Kramer and McLaughlin (2001)]. We observed that prey capture sequences performed by larval zebrafish consisted of an elementary swim pattern, separated by IBIs of decreasing duration (Figure 1). This is consistent with the relatively short interval observed between individual prey capture-related swim bouts (McElligott and O'Malley, 2005), and contrasts with the relatively long IBIs between routine swims in the absence of prey (Fuiman and Webb, 1988). Importantly, closed-loop experiments in which the larva was challenged with variable stimulus properties in between swims showed that the IBIs, and therefore reaction times, critically depended on position, size, and velocity of the updated target (Figures 5, 6). When approach was simulated by successively increasing target size and velocity after each swim [e.g. to (8°, 50°/s) after the 3rd swim], IBIs monotonically decreased between target-directed swims. By contrast, stimuli of comparable size and velocity (e.g., 8°, 40°/s) in isolation evoked avoidance swims directed away from the target when the larva was not in “prey capture mode” as judged by an unconverged eye position (Figure 4E). This suggests that the visuomotor system may transition into an internal state that facilitates prey capture behavior, during which visual feedback guides the sequence of target-directed motor patterns in rapid succession.

When is this visual feedback used to program the next discrete motor pattern in a sequence? Variable delay experiments in which the reappearance of the visual stimulus was timed to occur before, near or after the end of a swim revealed that the larva responded most rapidly to stimuli coinciding with swim termination. Notably, stimuli appearing before the end of the swim evoked a second swim with longer latencies, suggesting that the visuomotor system is less sensitive to visual feedback during execution of a swim bout. This could represent a form of movement-induced suppression of input processing, which has been observed in prey capture behavior, e.g., in the toad (Lock and Collett, 1979). In the primate visuomotor system, eye movement-induced “saccadic suppression” has been explained with a combination of corollary discharge and a forward visual masking mechanism (Wurtz, 2008). Functional experiments in the zebrafish may help to elucidate the neural substrates and mechanisms of a possible swim-induced suppression of visual processing during intermittent prey capture behavior.

Finally, we also observed that not only the timing of the visual feedback, but also its position had a significant effect on reaction times. A minimum of latencies was observed when the updated stimulus position was “head-on,” simulating a perfect alignment of the larva with the prey (Figure 6D). A qualitatively similar minimum of IBIs was also observed in the freely moving larvae (Figure 6E). This suggests that the larva is able to execute components of the prey capture sequence more rapidly when expected and perceived target positions overlap. Because this target-aligned configuration only occurs after the prey capture sequence has been initiated, when the target is centered in the binocular field of view, we speculate that a specific bilateral distribution of activity exists in the anterior-most region of the two tectal hemispheres that may trigger forward swims with minimal delay. By contrast, off-axis alignment of the prey during the sequence may require more processing time to program an asymmetric command component (A) in addition to the symmetric forward component (S) before the next swim is generated. The virtual reality techniques developed here may enable experiments using functional Ca^2+^ imaging and multiphoton-targeted patch-clamp recordings (Gabriel et al., 2012) to investigate the spatio-temporal distribution of activity underlying this complex form of visually guided behavior.

## Materials and methods

### Zebrafish maintenance

Zebrafish maintenance and breedings were carried out under standard conditions (Westerfield, 2007). Fertilized eggs were raised in embryo medium at 27°C under a 14/10 h light/dark cycle. Wildtype zebrafish larvae (ABTL) and *nacre* mutants (Lister et al., 1999) (6–8 days post-fertilization, dpf) were used. All procedures were performed according to the guidelines of the German animal welfare law and approved by the local administration.

### Freely moving larvae

Larvae were pre-selected based on whether they successfully performed prey capture sequences in the presence of paramecia during a ~5 min observation period under the dissection microscope. Selected larvae were transferred to a small arena (diameter 16 mm, height 5 mm) with opaque walls and a transparent bottom, filled with embryo medium to a height of 3–4 mm, to which a single paramecium was added. The chamber was illuminated from the top with white light using a goose neck lamp. Three arrays of infra-red LEDs (Kingbright, BLO-106) were mounted at a 45°-angle underneath the chamber to enable recording of the fish and paramecium under dark-field illumination. Prey capture sequences were recorded using a high-speed camera (AOS Imaging Systems, Model S-PRI 1039). A cold mirror was used to block visible light from the camera. Sequences were recorded at 250 or 500 frames/s. Recordings in which the larva or the prey touched the wall immediately before, during or after a prey capture sequence were discarded. All experiments were performed at room temperature, and bath temperature was observed to increase by no more than 2.5°C within 2 h.

### Partially restrained larvae

Larvae were preselected as described above. Selected larvae were anesthetized using 0.02% MS-222 in embryo medium for 5 min. The anesthetized larva was embedded in low-melting point agarose (4%) dorsal side up in the center of a quartz glass chamber (diameter 40 mm, height 25 mm, Hilgenberg, Germany), filled with a Sylgard base (~15 mm in height). After the agarose had set, the chamber was filled with embryo medium. Agarose around the head and tail was carefully removed using a scalpel, leaving only a thin collar surrounding the ear and the swim bladder. This allowed the larva to perform eye and tail movements. A diffusive material (E-color #216, Rosco, CA, USA) was attached to the outer wall of the chamber as a projection screen. Larvae were allowed to adapt to ambient light and embedding conditions for 15–30 min. After this period, the animal was monitored for spontaneous saccades and swim activity. Also, moving dot stimuli were shown to test the responsiveness of the animal. Larvae that did not respond with occasional directed swims or showed struggling behavior repeatedly in a ~30 min period were not used further. All recordings of motor activity in restrained larvae were performed at 500 frames/s.

### Visual stimulation

Visual stimuli were generated using custom-written programs in the Python based OpenGL VisionEgg software (Straw, 2008). Stimuli were projected onto the screen using a microprojector (Optoma Pico PK-102) at a refresh rate of 60 Hz. The visual scene consisted of a low spatial frequency surround of randomly positioned gray rectangles against a black background (nominal contrast ratio 16%). Small, rectangular targets (aspect ratio 2:1) moved against this stationary background at maximal contrast (“white”; gray level 255). Following swim detection (see below), the target and background moved synchronously at a pre-specified angular velocity (~400°/s) to a new angular position, to emulate retinal slip and the rotation of visual surround during an orienting turn of the larva. Subsequently the target resumed movement against the stationary background, depending on stimulus paradigm (see below).

Swimming and eye movements were recorded continuously at 500 frames/s during presentation of the moving target (“stimulus trial”), which was aborted after 56 s, if no response was observed. In order to control the stimulus in real time, the tail of the larva was projected onto a PSD (SiTek, 2L4-CP5) through a biconvex lens (*f* = 70 mm) via a 50/50 beam splitter in the acquisition path. Tail movement generated an oscillating signal in the PSD, which was sampled at 1 kHz (PCI-6259 board, National Instruments) and analyzed in real-time to detect the beginning and end of a swim bout. Threshold-crossing of the PSD-signal triggered the translation of the target and background to a pre-specified update location within ±10° of the visual field center. Stimulus presentation, high-speed video recording and acquisition of the PSD signal were synchronized using a common trigger signal. Using this system, updates of target and background could be implemented with an intrinsic delay of ~30–50 ms after the detection of a swim bout. Data acquisition and real-time updates of visual stimuli were controlled automatically by custom-written programs in LabVIEW.

### Stimulus paradigms

#### Optimization of prey-like stimulus

To test the effectiveness of small moving targets in triggering directed swims, we varied the size and velocity of the rectangular target, moving peripherally in the 30–50° range. Sixteen pairs of target size and velocity were chosen in the range 2–10° and 5–40°/s, respectively, similar to values measured during free prey capture sequences. Stimulus targets were shown randomly on the left or right side of the larva. During the experiment, the fish was observed to assume apparently different states. A state of prolonged rest was often followed by a state during which the larva performed periodic spontaneous saccades. Also, in some cases, struggle movements were observed that were followed by a prolonged resting state, lasting several minutes. Target-directed swim bouts were rarely observed following struggle swims or during the resting state. Therefore, trials were initiated during the state of spontaneous saccadic activity. A trial ended when the fish performed a swim bout or after 56 s, if no swim occurred. The response probability for different stimulus sizes and velocities was calculated for trials initiated under these conditions (Figure 4E).

#### Sequences of prey-like stimuli

To elicit motor sequences, first swims were evoked using a prey-like stimulus (2°; 20°/s) moving in the monocular visual field either between 30° and 50° or between 35° and 55° (interspersed trials on left or right side). Closed-loop visual feedback was implemented by translating the stimulus and background rapidly triggered by the detection of a swim bout. After stimulus/background translation, the stimulus resumed movement at a prespecified size and velocity toward the contralateral side of the visual field, which typically evoked another directed swim bout and eye convergence. Sequences of up to 6 target-directed swim bouts could be evoked by this method.

#### Kinematic analysis from high-speed video recordings

Automated image analysis of high speed movies was performed using custom written algorithms in LabVIEW and manual measurements on frames were performed in ImageJ.

#### Image analysis (free larvae)

High-speed recordings of freely moving larvae were saved as 8-bit grayscale movies and processed *post-hoc* to extract parametric information. First, a background image was subtracted from the movie, which highlighted the larva and the paramecium. Next, the 8-bit frames were scaled by histogram-equalization and converted to binary images using thresholding. Subsequently, binary morphological operations were performed that rejected small objects or those that touched the border of the image, and filled holes in binary objects. As a result, only the larva was visible as a binary object in each frame. Next, movie frames were automatically rotated such that the long axis of the larval binary object was aligned with the initial heading of the fish. Then, the binary object was converted to a distance map using the Danielsson distance mapping algorithm to reconstruct the midline of the larva. The distance map shows the distance of each pixel in a binary object to the nearest background pixel. The midline was reconstructed starting from the maximum pixel in the distance map, which is located near the center of the head. The positions of the maximum pixel in each image column were located iteratively toward the snout and tail and connected to obtain the midline of the larva. Next, the midline shape was approximated using a fit of six connected straight lines. The first segment was fit to the midline of the head (which was measured between the snout and the swim bladder). The remaining midline was fit using five line segments of equal length. Orientation of the fish (θ) was measured as the angle of the head segment of the midline in a global reference frame. Tail movement during swim bouts was quantified as the angle (γ_1_, …, γ_5_) between the individual tail segments and the body axis (defined as the heading direction of the first segment).

To track the position of the paramecium, the same morphological operations were performed on the binary movie, but with a processing step that rejected large objects, leaving the paramecium as the only object. After manual selection of the paramecium in the first frame, the algorithm tracked its trajectory automatically for all subsequent frames. Furthermore, the fish-target angle (ϕ) and the fish-target distance (d) (i.e., the distance between the center of the head segment and the centroid of the paramecium) were measured on a frame-by-frame basis in which the binary images of the fish and the paramecium were overlaid. The distance traveled during a swim bout was calculated as the difference between the fish-target distance before and after the swim.

High-speed recordings of freely moving larvae covered a larger field of view, which precluded an automatic analysis of kinematic parameters of small details. Therefore, we manually measured ipsilateral and contralateral eye angles with respect to the body axis in individual frames before and after a swim bout. Also, angular size and angular velocity of the prey in the interval between two swims were manually determined by measurements in individual frames. We also manually measured fish-target angle in the frames before and after a swim bout as well as change in orientation to corroborate our automated analysis.

#### Image analysis (restrained larvae)

For minimally restrained larvae, we could use the image analysis algorithm designed for freely moving larva after minor modifications. Since there was no reference frame for background subtraction, we used a linear remapping of the histogram to reduce background pixel values and increase fish pixel values. The linear remapping factor was adjusted empirically for each experiment. Subsequently, the gray scale image was binarized using fixed threshold values. Binary morphological operations identical to the ones mentioned earlier were performed to ensure that the image consisted of one object i.e., the larva. Distance mapping and midline extraction procedures were also the same as described above.

In addition to the tail segment angles (γ_1_, …, γ_5_), the algorithm also measured ipsilateral and contralateral eye angles automatically for restrained larvae. The algorithm prompted the user to draw a region of interest around the head which included both eyes. This region of interest was then extracted and an adaptive negative thresholding subroutine was used to detect the two eyes. Subsequently, binary morphological operations were performed and two binary objects were obtained. Each eye was fitted with an ellipse and the angle between the major axis of the ellipse and the body axis of the fish was recorded as the eye angle.

#### Fourier analysis of swim bouts

In order to investigate the frequency composition for each swim bout, we performed discrete Fourier analysis using a temporal window around the swim traces. The length of this window was set to 150 ms for freely moving larva and 300 ms for restrained larva. A Bessel band-pass filter was then applied to the extracted individual waveforms, each containing deviations of one of the five tail segments with respect to the body axis (γ_1_, …, γ_5_). The low cut-off frequency of the filter was set to 0.5 Hz to eliminate the effect of a slow drift in the tail that is often observed following swim bouts. The high cut-off frequency of the filter was set to 100 Hz to eliminate high frequency noise that may result from frame-to-frame jitter in tail angle measurements. A discrete Fourier transform (DFT) was then applied to these filtered waveforms. A DFT operation on a time series resulted in a two-sided complex spectrum. This spectrum was then converted to a two-sided amplitude spectrum.

$$\begin{array}{l} X_{k}=\sum_{n=0}^{N-1}x_{n}\;\cdot \;e^{-i2\pi n\frac{k}{N}},\text{ where }k=0,\;1,\;2,\;\ldots ,\;N-1\\ \text{ (Discrete Fourier Transform)}\\ A_{k}=\frac{X_{k}}{N},\text{ where }k=0,\;1,\;2,\;\ldots ,\;N-1\\ \;(\text{Two-sided complex spectrum}) \end{array}$$

Since the DFT spectrum is conjugate symmetric, this two-sided spectrum was then converted to a single-sided spectrum by using the first (*N*/2–1) components.

$$\begin{array}{l} B_{k}=A_{0},\text{ for }k=0\text{ else},\\ B_{k}=\sqrt{2}\;\cdot \;A_{k},\text{ for }k=1,\;2,\;\ldots \;N/2-1\\ \;(\text{Single-sided RMS spectrum}) \end{array}$$

The magnitude of this complex single-sided RMS spectrum was used to obtain an RMS amplitude spectrum.

$$\begin{array}{l} B_{mag}=\left|B_{k}\right|,(\text{Magnitude for the RMS spectrum}) \end{array}$$

This resulted in a single-sided amplitude spectrum for each individual tail segment. Subsequently, we summed these individual spectra to obtain one spectrum for each swim. Since the frequency resolution for a spectrum is limited by the sampling frequency and the Nyquist criterion, we used a spline interpolation algorithm to improve our estimate of the magnitude at different frequencies in the spectrum. Further, to assign directionality to each swim, the temporal traces of all five tail angles γ_1_, …, γ_5_ were averaged and subsequently integrated. The sign of this integral was subsequently multiplied with the amplitude of the low frequency peak in the sum spectrum to obtain a “DI” for each swim.

$DI=sign\left[\int_{0}^{t}dt\frac{1}{5}\sum_{i=1}^{5}\gamma_{i}(t)\right]\times \frac{1}{3}\sum_{k=3}^{5}B_{mag_{k}}$, where γ_*i*_ is the angle for *i*th tail segment and *B*_mag_ is the value of the sum spectrum at *k*th frequency, here yielding an average peak amplitude between 3 and 5 Hz. Spectral analysis and the computation of directional index was performed using Labview8.6 (National Instruments).

### Conflict of interest statement

The authors declare that the research was conducted in the absence of any commercial or financial relationships that could be construed as a potential conflict of interest.

## Supplementary material

Movie S1 **Movie of a sequence of swim bouts performed by a 6 dpf larval zebrafish during prey capture.** Movie recorded at 250 frames/s and played back in real time.

Movie S2 **Same movie as in S1, played back at 30 frames/s**.

Movie S3 **Movie of a restrained larval zebrafish responding to a stimulus sequence with directed swim bouts and eye convergence.** Movie recorded at 500 frames/s and played back in real time. The stimulus frames were generated and recorded using VisionEgg software and overlaid with the movie in LabVIEW.

Movie S4 **Same movie as in S3, played back at 60 frames/s**.
